# HLA-driven variability in human cytomegalovirus immune recognition: Epitope predictions from a kidney transplant Cohort

**DOI:** 10.1371/journal.pone.0360632

**Published:** 2026-10-15

**Authors:** Mirella Dara Moreno Rosado, Tatiana Chavez-Arias, Obert Marín-Sánchez, Ricardo J. Juarez Zevallos, Alexis Germán Murillo Carrasco, César A. Ortiz Rojas, Yuma Ita-Balta, Ruy Diego Chacón Villanueva

**Affiliations:** 1 Facultad de Ciencias Biológicas, Universidad Nacional Mayor de San Marcos, Av. Carlos Germán Amezaga, Lima, Peru; 2 Pathogen Genetics Research Group (PATHO-GEN), Organization for Medical Innovation and Collaboration for Sciences (OMICS), Lima, Peru; 3 Immunology and Cancer Research Group (IMMUCA), Organization for Medical Innovation and Collaboration for Sciences (OMICS), Lima, Peru; 4 Facultad de Medicina Humana, Universidad Católica de Santa María, Arequipa, Peru; 5 Área de Histocompatibilidad y Biología Molecular, Hospital Nacional Carlos Alberto Seguin Escobedo, Red Asistencial Arequipa, Seguro Social de Salud (EsSalud), Arequipa, Peru; 6 Facultad de Medicina, Universidad Nacional Mayor de San Marcos, Lima, Peru; 7 Escuela de Postgrado, Universidad Católica de Santa María, Arequipa, Peru; Sefako Makgatho Health Sciences University, SOUTH AFRICA

## Abstract

**Background:**

Human cytomegalovirus (HCMV) infection causes life-threatening complications in transplant recipients. Despite a seroprevalence exceeding 90% in Peru, the influence of HLA diversity on HCMV immune recognition remains unexplored in the South American region. We aimed to characterize the HLA allele distribution in a Peruvian cohort and evaluate its impact on the predicted HCMV antigen-presentation landscape.

**Methods:**

HLA genotyping (loci A, B, C, DRB1, DQA1, DQB1) was performed in 562 kidney transplant candidates and related donors. Common allele groups (AF5%) were assigned 4-digit resolution proxies based on local prevalence. Linear T-cell epitopes from the immunodominant antigens 65 kDa phosphoprotein (pp65), Immediate early protein 1 (IE1), and Envelope glycoprotein B (gB) were predicted using high-performance algorithms. Allelic capacity to strongly bind antigenic peptides was quantified using the Predicted Strong Binder Density (PSBD).

**Results:**

We identified 16 HLA-I and 10 HLA-II common allele groups; 92% of their 4-digit proxies are widely distributed in SAM, while C*15:12 emerged as a potentially endemic variant in Peru. Screening yielded 954 HLA-I candidate epitopes, including 169 that correspond to previously reported IEDB epitopes with potential for immunotherapy. PSBD analysis revealed distinct predicted antigen-presentation profiles across the common HLA variants identified.

**Conclusion:**

Our findings provide updated Peruvian HLA data and define regional HCMV antigen-presentation profiles based on predicted MHC-peptide binding affinity (PSBD). Further experimental validation of these high-confidence candidate epitopes and HLA-HCMV association studies will be necessary to determine their immunogenicity and potential utility for immune monitoring and future immunotherapeutic applications across South American populations.

## Introduction

Human cytomegalovirus (HCMV), defined as *Cytomegalovirus humanbeta5* according to the ICTV 2026 release (MSL #41) report [1], is the prototype member of the β-herpesvirus subfamily of herpesviruses, thus establishing a lifelong infection like all herpesviruses [2]. Although this infection is generally asymptomatic, it can lead to life-threatening complications in immunocompromised individuals, such as Solid Organ Transplant (SOT) and Hematopoietic Stem Cell Transplant (HSCT) recipients, in whom it remains the most common and serious opportunistic infection [3].

HCMV is globally widespread, and its seroprevalence appears to be correlated with ethnicity, socioeconomic status, and education level [4]. Overall, in Latin American countries, seroprevalence in adults ranges from 59.1% to 91.3% [4], while in Peru, high HCMV seroprevalence has been reported in post-heart-transplant patients (63%) [5] and blood donors at public health centers (91.7%, 97%) [6,7].

HCMV control relies on a coordinated response of both innate and adaptive immunity. Within adaptive immunity, CD8 T cells restrict viral replication in most tissues during acute infection, whereas CD4 T cells are primarily required for controlling persistent infection, particularly in sites of viral persistence and shedding such as the salivary glands [8]. As different HLA alleles encode distinct peptide-binding repertoires [9,10], specific HLA variants called HCMV immune-dominant alleles, which include A*02:01 and B*07:02, have been associated with resistance to HCMV reactivation and more effective antigen presentation [11]. Furthermore, HLA diversity is influenced by populations’ exposure to pathogens; consequently, HLA frequency variations between populations provide population-level resistance or susceptibility to several diseases [12]. However, existing HLA-HCMV clinical research, both in vitro and retrospective, predominantly focuses on Asian and European cohorts [13–16].

Besides the fact that typed HLA alleles remain unevenly represented across different geographical regions [17], the coverage population of databases like the Allele Frequency Net Database (AFNDB) and the Immune Epitope Database (IEDB) may not show an accurate representation of HLA diversity in South American (SAM) populations due to small sample sizes and bias towards specific ethnic groups [18]. This underrepresentation limits the identification of population-relevant T-cell epitopes and may consequently hinder the development and implementation of HLA-informed applications, including HCMV-specific T-cell therapy (CMV-TCT) [19] and immune monitoring of antigen-specific cellular responses, which is important for risk stratification and personalization of patient treatment [20,21]. Because HLA typing is routinely performed as part of donor–recipient matching, kidney transplant cohorts provide a valuable source of immunogenetic data to address these knowledge gaps.

Given this context, we aimed to characterize the HLA allele distribution in a Peruvian Kidney transplant cohort and evaluate how these variants could shape the predicted HCMV antigen-presentation landscape, based on the HLA class I genotypes. Our findings provide a framework for future experimental validation of candidate epitopes and HLA-associated reactivity. Because the present study relies exclusively on computational predictions, the results should be interpreted as estimates of potential antigen presentation rather than direct measures of T-cell immune responses, immunogenicity, or clinical protection.

## Methods

### Ethical approval

This study received ethical approval from the Institutional Research Ethics Committee of the Arequipa Healthcare Network – EsSalud (Approval Letter No. 000010–2026-UCIYD-GRAAR), issued on January 14, 2026. Given that this research involved a secondary analysis of large-scale, fully anonymized data, the requirement for obtaining informed consent from participants was waived in accordance with national and institutional ethical guidelines.

### HLA genotyping

HLA genotyping was performed at 6 loci: HLA-A, -B, -C, -DRB1, -DQA1, and -DQB1, in kidney transplant candidates (*n* = 131) and their respective donors (*n* = 431) at the Laboratorio Central del Hospital Nacional Carlos Alberto Seguín Escobedo in Arequipa, Peru, from 2019 to 2024. HLA allelic groups were determined using the Polymerase Chain Reaction-Sequence Specific Oligonucleotide Probe (PCR-SSO) technique with One Lambda™ LABScan™ HLA kit (ThermoFisher Scientific), and the results were obtained using Luminex® xPONENT® software. Except for HLA-B in one patient and HLA-C in another patient, the 6 loci were analysed in all samples. Thus, a total of 562 HLA-A, -DRB1, -DQA1, and -DQB1 genotypes and 561 HLA-B and HLA-C genotypes in this study. These protocols are routinely implemented in the hospitals mentioned above. For this study, retrospective data were collected secondarily and fully anonymized, with no possibility of identifying any study participants. The results were retrieved from the digital reporting system on January 15, 2026.

### Estimation of common HLA alleles

We determined the representativeness of the Peruvian population’s HLA alleles by applying an allele frequency (AF) threshold of ≥5% to define common alleles or allele groups, ensuring the prediction of regionally relevant immunogenic epitopes and the exclusion of rare alleles whose impact on regional population immune response may be limited [18]. We complemented this with a previous classification of HLA alleles based on AFs [17] and organized allele groups in three non-mutually exclusive groups, as follows: common (AF ≥ 5%), frequent (AF ≥ 10%), or very frequent (AF ≥ 20%) groups.

Since PCR-SSO provides intermediate-resolution typing and does not determine haplotypic phase, 4-digit proxies for common HLA class I and II allele groups were assigned by selecting the highest AFs from 4-digit-resolution data from Peruvian cohorts available in the literature and the AFNDB [22] (S1 Material). Reference HLA allele group diversity was determined from IPD-IMGT/HLA 3.63.0 (2026-01-14) [23], using Python v3.12.4 (Table 1). Accordingly, downstream analyses based on inferred four-digit alleles should be interpreted as population-level estimates of antigen presentation.

**Table 1:** Locus-Specific Allele Groups and High-Resolution (4-Digit) Alleles.

*Locus (Reported Allele Groups)*	*Observed Allele Groups (n)*	*4-digit resolution Alleles (n)*
HLA-A (n = 21)	n = 16 (A*01, A*02, A*03, A*11, A*23, A*24, A*25, A*26, A*29, A*30, A*31, A*32, A*33, A*34, A*66, A*68)	n = 10 (A*02:11, A*02:12, A*02:13, A*02:22, A*02:33, A*02:39, A*68:01, A*68:02, A*68:100, A*68:17)
HLA-B (n = 36)	n = 24 (B*07, B*08, B*13, B*14, B*15, B*18, B*27, B*35, B*37, B*38, B*39, B*40, B*41, B*44, B*45, B*47, B*48, B*51, B*52, B*53, B*55, B*57, B*58, B*81)	n = 28 (B*15:04, B*15:17, B*15:63, B*35:01, B*35:04, B*35:05, B*35:06, B*35:09, B*35:10, B*35:15, B*35:19, B*35:21, B*35:22, B*35:232, B*38:157, B*39:05, B*39:09, B*39:12, B*39:13, B*39:14, B*40:04, B*40:08, B*40:64, B*45:01, B*51:13, B*51:154, B*51:31, B*58:01)
HLA-C (n = 14)	n = 13 (C*01, C*02, C*03, C*04, C*05, C*06, C*07, C*08, C*12, C*14, C*15, C*16, C*17)	n = 1 (C*05:29)
HLA-DRB1 (n = 13)	n = 13 (DRB1*01, DRB1*03, DRB1*04, DRB1*07, DRB1*08, DRB1*09, DRB1*10, DRB1*11, DRB1*12, DRB1*13, DRB1*14, DRB1*15, DRB1*16)	n = 11 (DRB1*01:54, DRB1*03:70, DRB1*04:02, DRB1*04:172, DRB1*04:174, DRB1*04:56, DRB1*08:07, DRB1*09:01, DRB1*09:04, DRB1*11:54, DRB1*13:04)
DQA1 (n = 6)	n = 6 (DQA1*01, DQA1*02, DQA1*03, DQA1*04, DQA1*05, DQA1*06)	n = 5 (DQA1*02:01, DQA1*03:01, DQA1*04:01, DQA1*05:01, DQA1*06:01)
DQB1 (n = 5)	n = 5 (DQB1*02, DQB1*03, DQB1*04, DQB1*05, DQB1*06)	n = 1 (DQB1*06:49)
Total	77	56

Note: Reported Allele Groups based on IPD-IMGT/HLA Release 3.63.0. High-resolution alleles were computationally aggregated to identify unique 2-digit allele groups.

### Epitope prediction

Due to the lack of haplotypic phase information required for reliable reconstruction of HLA-DQ α/β heterodimers, accurate HLA class II epitope prediction was not feasible. Therefore, epitope prediction was restricted to HLA class I alleles. For this purpose, the immunodominant HCMV antigens, 65 kDa phosphoprotein (pp65; UniProt ID: P06725), Immediate early protein IE1 (IE1; UniProt ID: P13202), and Envelope glycoprotein B (gB; UniProt ID: P06473) [24,25], the proteins containing most HCMV T-cell epitopes and MHC ligands reported in the IEDB [26], were selected for linear T-cell epitope prediction. Peptide lengths of 8, 9, 10, and 11 amino acids, the consensus framework, and the updated versions of software described by Requena et al. [18]: NetMHCpan 4.1 and MHCflurry v.2.1.5 [27–29] were adopted.

Candidate HLA-I epitopes were subsequently classified into Strong Binders (SB) and Weak Binders (WB) according to software-recommended thresholds for HLA-I epitopes (SB: %Rank ≤ 0.5 or IC50 ≤ 50 nM; WB: %Rank ≤ 2 or IC50 < 500 nM). The consensus framework was applied to both epitopes and SB.

In addition, HLA-I candidate epitopes predicted to bind at least 35% of the analyzed HLA-I molecules (≥6 of 16 alleles) were classified as promiscuous. This threshold was chosen to balance prediction stringency and population coverage, given that candidate epitopes were already filtered using consensus predictions from two independent algorithms. Finally, sequences identical to previously validated IEDB epitopes (positive outcomes in T-cell or MHC binding assays) were labeled as experimentally validated epitopes, while unmatched sequences were designated as novel epitopes.

### Epitope density and promiscuity metrics

To enable standardized comparisons across the three HCMV proteins, the numbers of predicted strong binders (SB) and candidate epitopes (WB + SB) were normalized to the source protein length, hereafter referred to as Predicted Strong Binder Density (PSBD) and Predicted Candidate Epitope Density (PCED), respectively. In addition, epitope promiscuity was estimated using the Tepi:Nepi ratio (total HLA–peptide binding events divided by the number of unique candidate epitopes), representing the average number of HLA alleles predicted to bind each unique peptide [30].

### Repertoire size allele groups

HLA alleles were stratified into wide-, intermediate-, and narrow- repertoire size groups, following the conceptual framework of Paul et al. [31], where Repertoire Size is defined as the fraction of all possible peptides within a dataset that an allele is capable of binding below a given affinity threshold. Because the consensus framework adopts a conservative prediction strategy, repertoire size estimates may be lower than those obtained using individual prediction algorithms. Therefore, repertoire size thresholds were established using the Jenks natural breaks method to identify natural groupings within the observed distribution, enabling a relative ranking of predicted allele-specific repertoire size. These categories should be interpreted as descriptive classifications within the present dataset and not as biologically validated thresholds.

### Epitope annotation and mapping

Epitope features, including structural, functional, and Post-Translational Modifications (PTMs), were retrieved via UniProt REST API [32] (Supplementary Methods) and cross-referenced with predicted coordinates; single-coordinate features were treated as residue-level annotations. 3D structures for IE1 (6TGZ) and gB (5CXF) were sourced from PDB, while pp65 was modeled via I-TASSER [33]. Finally, candidate and experimentally validated epitopes were visualized on these structures using UCSF Chimera [34]. Structural mapping was performed as an exploratory visualization tool to contextualize the spatial distribution of predicted and previously validated epitopes. These analyses were not intended to infer functional consequences of epitope localization or immune recognition mechanisms.

To provide additional biological context for the predicted epitopes, homologous HCMV protein sequences (Query coverage ≥ 70%) were retrieved from GenBank using BLASTP searches. Multiple sequence alignments were generated using MAFFT [35] and the residue variability at each position was measured by Shannon Entropy [36], calculated with the Shannon Entropy-one online tool (https://www.hiv.lanl.gov/content/sequence/ENTROPY/entropy_one.html) across pp65, IE1, and gB proteins. Position-specific conservation scores were then computed as $\text{1}-\frac{H}{ln(\text{2}\text{0})}$, where H is the Shannon entropy.

Experimentally validated IEDB epitopes, predicted epitopes, functional domains, and annotated post-translational modification sites were subsequently mapped onto linear protein representations.

## Results

### Study cohort

The study included a total of 562 individuals, comprising 131 transplant candidates (Case group) and 431 family members evaluated as potential living donors (Family group). Overall, the median age was 47 years (range: 11–85 years). Patients in the Case group were younger, with a median age of 38 years (range: 18–57 years), whereas individuals in the Family group had a median age of 50 years (range: 18–85 years). Regarding sex distribution, females represented a slight majority in the overall population (52.0%, *n* = 291), while males accounted for 48.0% (*n* = 271). In the Case group, 56.0% (*n* = 74) were female, and 44.0% (*n* = 57) were male, while the Family group exhibited an approximately equal distribution between females (50.3%, *n* = 217) and males (49.7%, *n* = 214). Among the potential donors, the most frequent group was first-degree relatives, particularly mothers (*n* = 107), fathers (*n* = 92), brothers (including half-brothers; *n* = 108), and sisters (including half-sisters; *n* = 99). A smaller proportion of evaluations involved more distant relatives, such as uncles and aunts (*n* = 13), offspring (*n* = 6), and other family members (*n* = 6).

### HLA-related population profile

From all described samples (*n* = 562), we were able to characterize over 99.8% of the alleles. This study only lost information on alleles for HLA-B in one patient and HLA-C in another patient (S1 Table). No imputation was performed for these missing values.

In sum, 53 HLA class I (-A, -B, and -C) and 24 class II (-DRB1, -DQA1, -DQB1) allele groups were identified. Among them, we selected the 16 HLA-I common allele groups for high-resolution assignment (S2 Table) and downstream analysis. This included frequent and very frequent allele groups (Fig 1). Notably, the proportion of very frequent allele groups was higher in HLA-II loci, with 7 families meeting the threshold. Particularly, high-resolution typing was obtained for 398 allele assignments, allowing the identification of 56 distinct HLA alleles at the four-digit level (S3 Table). At low resolution, HLA-B was the most diverse locus in our cohort, with 24 HLA-B allele groups. These included B*35, for which 10 distinct 4-digit-resolution alleles were identified. Meanwhile, DQA1 and DQB1 showed the lowest number of allele groups, as expected due to the total HLA-II known allele groups (Table 1). Detailed HLA frequency distributions per locus are shown in S1 Fig.

**Fig 1:**
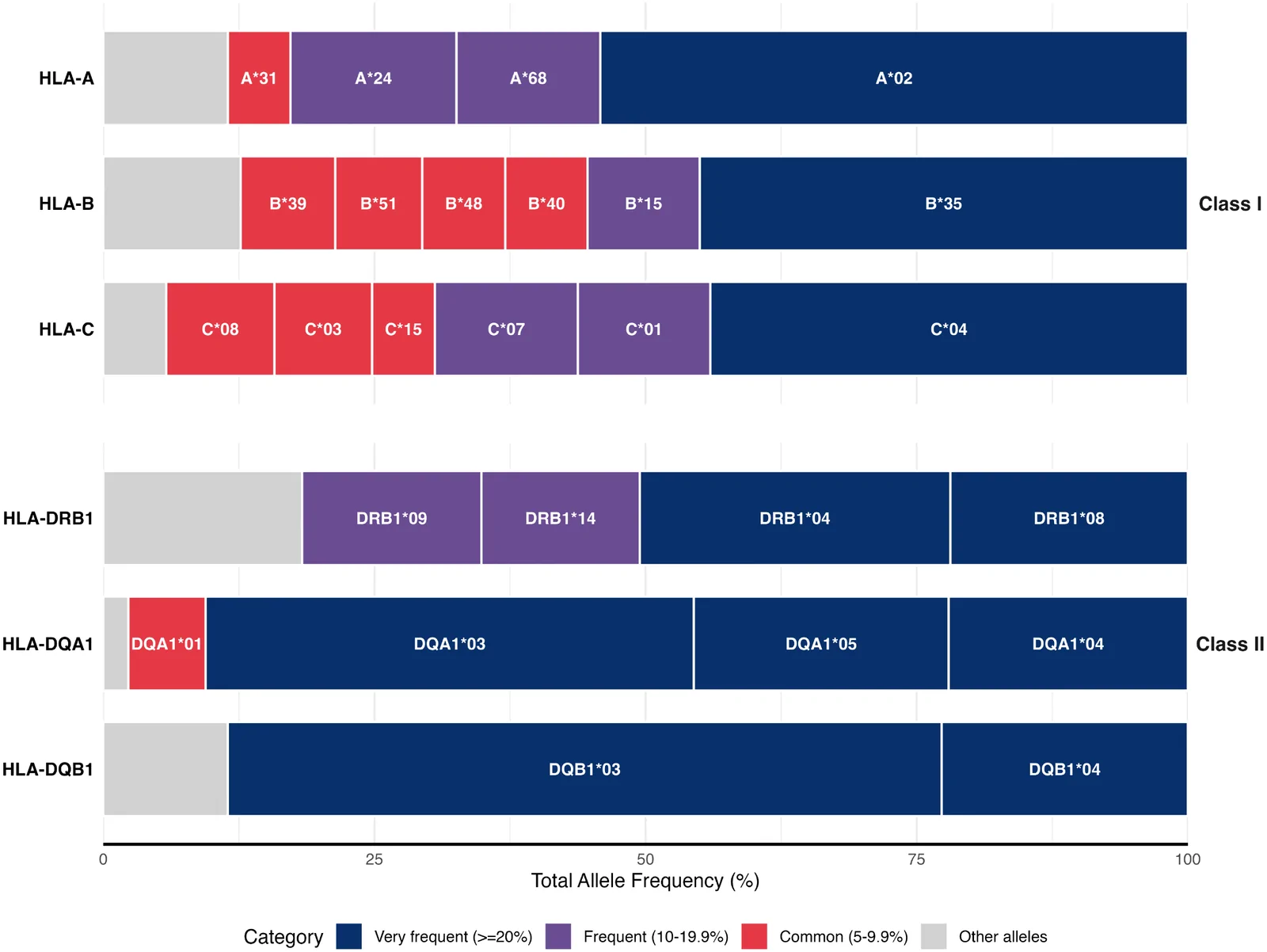
Distribution of common HLA allele group frequencies in the studied cohort. Horizontal stacked bars show the cumulative AF per locus for HLA class I (HLA-A, -B, -C) and class II (HLA-DRB1, -DQA1, -DQB1). Complete allele frequency data are provided in S1 Table, and the full distribution of all allele groups per locus is shown in S1 Fig. AF, allele frequency.

### HCMV novel T-cell epitope candidates across gB, pp65 and IE1

A total of 1593 HLA-peptide binding events and 954 HLA-I candidate epitopes (Nepi) were predicted across 3 HCMV immunodominant proteins, gB (906 aa), pp65 (561 aa) and IE1 (491 aa) (Table 2), and 16 common HLA alleles in the studied cohort (S4 Table). None of the candidate epitopes were shared among the proteins.

**Table 2:** Comparative analysis of predicted HLA class I epitopes among HCMV proteins.

Parameter	gB (906 aa)	pp65 (561 aa)	IE1 (491 aa)	Overall
**HLA-peptide binding events (Tepi)**	776 (48.7%)	471 (29.6%)	346 (21.7%)	1593 (100%)
Strong Binding events	124 (52.5%)	60 (25.4%)	52 (22.0%)	236 (100%)
Weak Binding events	652 (48.0%)	411 (30.3%)	294 (21.7%)	1357 (100%)
**Candidate epitopes (Nepi)**	469 (49.2%)	288 (30.2%)	197 (20.6%)	954 (100%)
Strong Binders (SB)	94 (50.3%)	52 (27.8%)	41 (21.9%)	187 (100%)
SB among candidate epitopes (%)	20	18.1	20.8	19.6
**Experimentally validated epitopes**	19 (4.1%)	119 (41.3%)	31 (15.7%)	169 (17.7%)
**Derived Metrics**				
Tepi:Nepi Ratio	1.7	1.6	1.8	1.7
Predicted Candidate Epitope Density (PCED) (%)	51.8	51.3	40.1	–
Predicted Strong Binder Density (PSBD) (%)	10.4	9.3	8.4	–
**HLA alleles**				
Highest epitope count	HLA-C*07:02 (97)	HLA-C*07:02 (60)	HLA-C*01:02 (36)	–
Lowest epitope count	HLA-B*48:01 (0)	HLA-B*48:01 (2)	HLA-B*48:01 (0)	–

*Note. Values in parentheses indicate percentages relative to the overall number of binding events or candidate epitopes, as appropriate. —, not applicable.*

Nearly half of the binding events originated from gB-derived peptides (48.7%), followed to a lesser extent by those of pp65 (29.6%) and IE1 (21.7%). The same hierarchy, gB > pp65 > IE1, was observed in the total count of candidate epitopes or SB, together with the PCED and PSBD metrics (Table 2). Conversely, the proportion of predicted epitopes that qualified as SB was similar in gB (20.0%) and IE1 (20.8%), but slightly lower in pp65 (18.1%).

### Experimentally validated epitopes

A sum of 169 HLA-I candidate epitopes (17.7%) were found to overlap with previously validated epitopes, most of them derived from pp65 (potential binding positions are shown in S2 Fig.).

### Analysis of promiscuous epitopes

The three evaluated antigens showed nearly identical Tepi:Nepi Ratio, indicating that each candidate epitope was predicted to bind, on average, to 1.7 HLA-I alleles, regardless of the protein from which it was derived.

Applying the established promiscuity threshold (≥35%, equivalent to ≥6/16 HLA-I molecules), we identified a total of 28 promiscuous epitopes (Fig 2). Despite the Tepi:Nepi Ratio being pretty much the same across the three proteins, the PCED and PSBD values followed the hierarchy (gB > pp65 > IE1)

**Fig 2:**
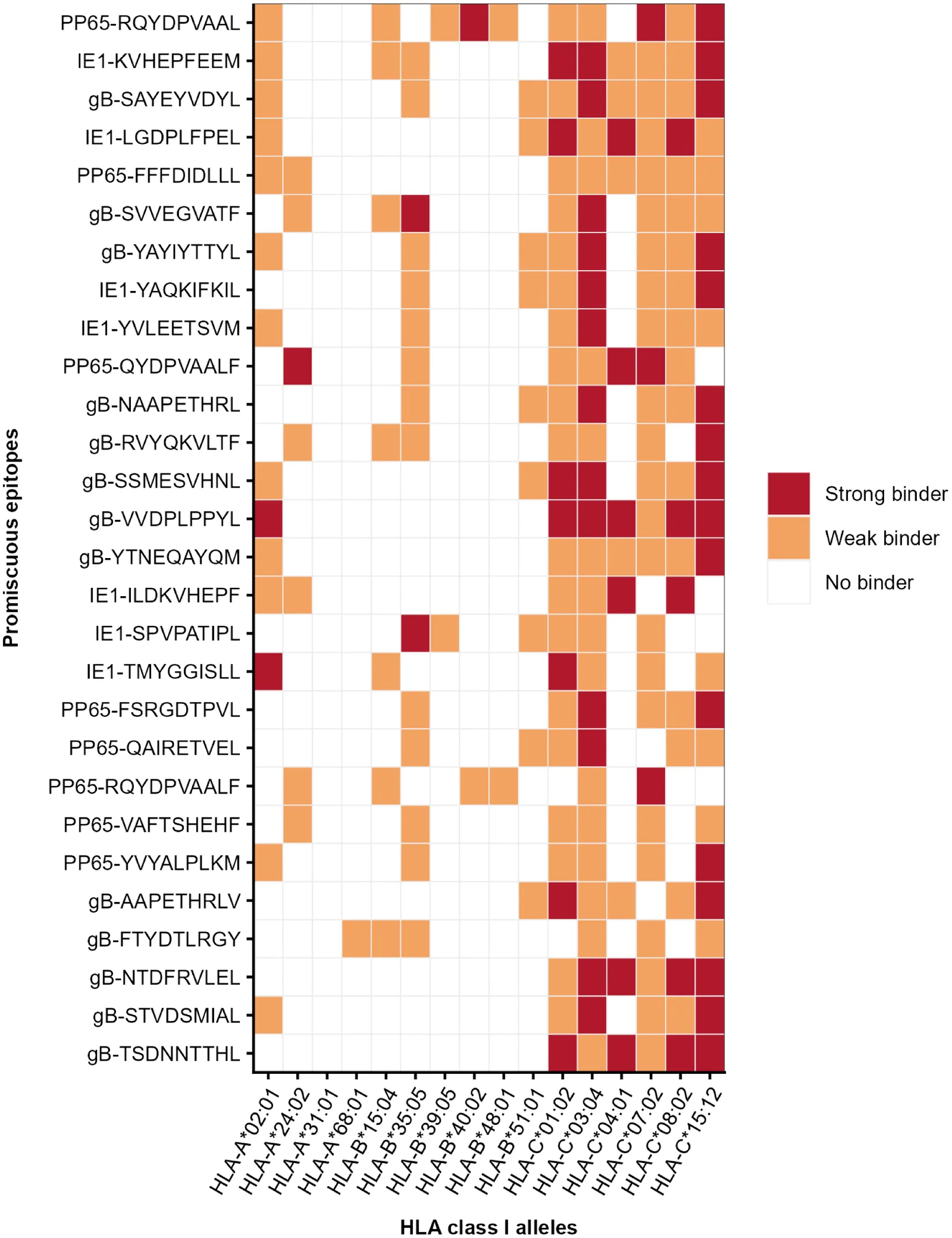
Heatmaps of predicted epitope promiscuity. Peptides predicted to bind a minimum of 6 of the 16 HLA-I alleles evaluated (≥35% threshold). Note that strong-binding interactions are represented in red, weak-binding in orange, and the absence of predicted binding in white. Binding criteria: Class I—strong binders (%Rank ≤ 0.5; IC50 < 50 nM) and weak binders (%Rank ≤ 2; IC50 < 500 nM).

Promiscuity of candidate epitopes did not reflect higher binding strength. Although pp65-RQYDPVAAL was predicted as the most promiscuous HLA-I epitope, binding 10/16 HLA-I molecules evaluated, it counted as SB for 3 HLA-I molecules, while gB-VVDPLPPYL counted as SB for 6 HLA-I molecules, resulting in the epitope with the highest count of SB (S5 Table).

### Repertoire Size HLA profile

The analysis of repertoire size revealed marked differences in the predicted HCMV antigen-presentation capacity across HLA-I alleles (Fig 3A). Half of the evaluated alleles fell into the wide-repertoire-size category (1.5–2.47%) (Fig 3B), with -C*07:02 at the top of the list, though it shows no strong binding to IE1. Notably, all evaluated HLA-A alleles resulted in wide repertoire sizes, except for -A*24:02, classified herein as an intermediate-repertoire-size allele (0.82% − 1.25%). In contrast, the narrow-size-repertoire (0.03–0.5%) group was comprised exclusively by HLA-B alleles, with B*15:04 and B*48:01 showing no strong binding at all. The remaining HLA-B alleles were classified within the intermediate-repertoire-size group, whereas only B*40:02 belonged to the wide-repertoire-size group.

**Fig 3:**
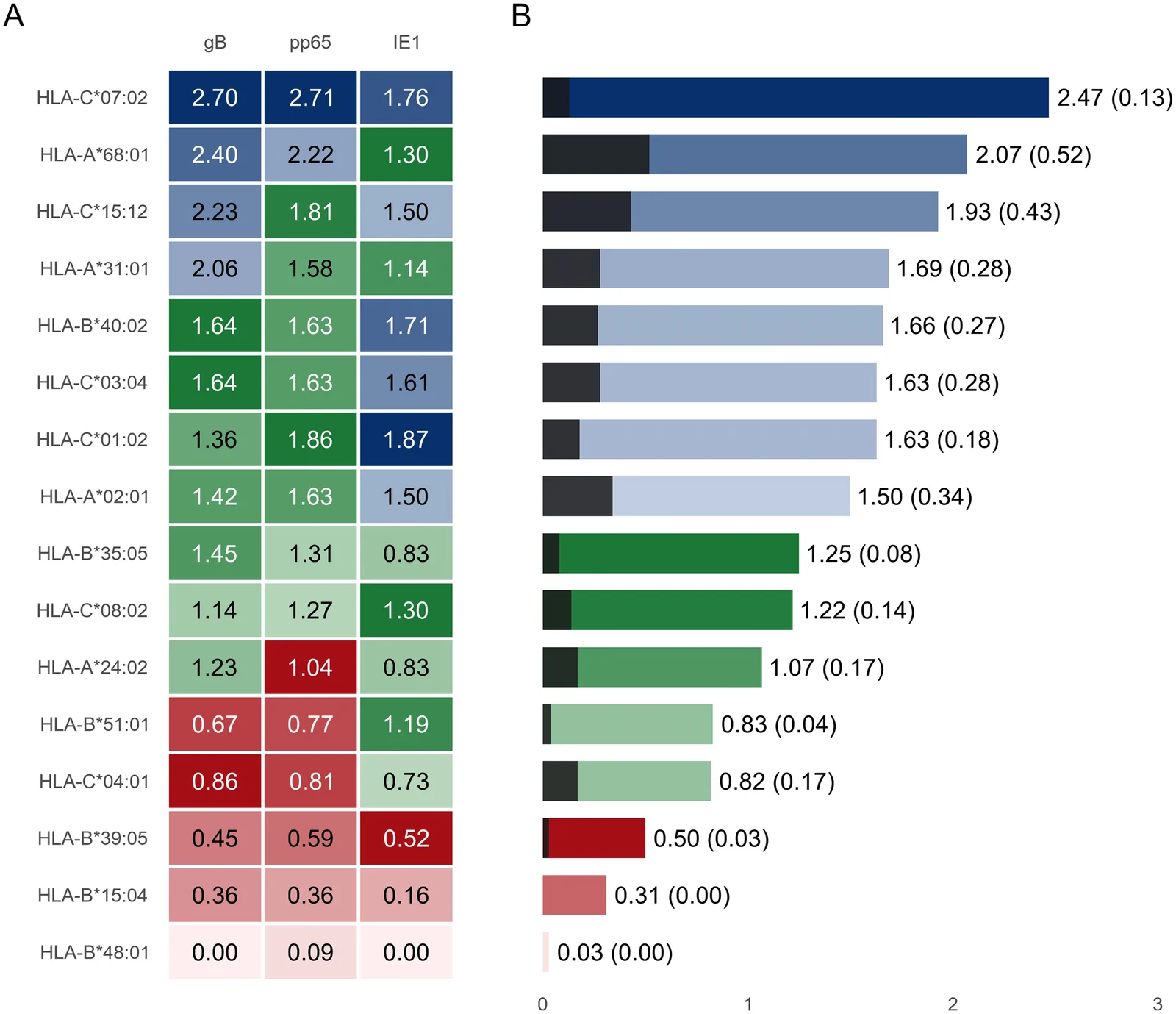
Repertoire size of HLA alleles against HCMV antigens. (A) Repertoire size (%) per antigen (gB, pp65, IE1) for each HLA allele, ordered by descending overall repertoire size. (B) Overall repertoire size (%) and strong-binder (SB) repertoire size (%, black overlay) per allele; values shown as total (SB). Color indicates the allele’s repertoire size group for that column, narrow (red), intermediate (green), wide (blue), with shade intensity proportional to value within the group.

## Discussion

We present the first assessment of HLA allelic diversity and the predicted HCMV antigen-presentation landscape in a Peruvian transplant cohort. To address the decade-long lack of updated regional high-resolution data in AFNDB [18,37–41], we restricted predictions to common HLA-I alleles (2019–2024). This minimized potential bias and enabled high-resolution designations that supertyping could not resolve. The cohort’s diversity reflected global patterns [IPD-IMGT/HLA Release 3.63.0–2026−01] [23,42], with HLA-B exhibiting the highest diversity of HLA groups and the HLA-II loci (-DQA1 and -DQB1) the lowest.

Twenty-four of the 26 analyzed alleles are common in SAM populations, including the regionally frequent B*15:04 and the universally prevalent alleles A*02:01, A*24:02, and DQA1*03:01 [17]. In contrast, C*08:02 is only common in Brazil and near-common (AF = 4.92%) in Colombia [22]. As a novelty, C*15:12 appears potentially endemic to Peru; while exceptionally rare (<0.001%) in German and US cohorts, it reached a 4.28% AF in the Peruvian source cohort [22,37]. In addition, the wide-repertoire-size status of this allele (1.93%) (Fig 3) suggests that regional variants may contribute to distinct HCMV antigen-presentation profiles.

The average Tepi:Nepi ratio was used as a measure of epitope promiscuity per antigen. Our analysis of the three most antigenic HCMV proteins yielded an average value of 1.7, substantially lower than the average ratio of 5.9 reported for hepatitis B virus (HBV) in the Botswana population. Nevertheless, several promiscuous epitopes were identified in our cohort, including gB-YAYIYTTYL and pp65-QYDPVAALF, which have been previously reported as targets of CTL responses and have been explored in adoptive immunotherapy strategies through polyepitope integration and multi-ethnic recognition [26,43,44]. In particular, the extensively characterized pp65-QYDPVAALF interacts with HLA-A24:02 and HLA-C04:01 [26,45–53] and has been associated with cytokine production and cytotoxic T-cell responses in previous experimental studies, supporting its relevance as a candidate target for TCR-engineered T-cell therapies [26,54,55].

Applying a consensus of NetMHCpan (4.1) and MHCflurry (v2.1.5), both state-of-the-art tools trained on MHC binding and ligand elution data [56], we identified high-confidence regional candidate epitopes for future validation and potential evaluation in HCMV immune-monitoring strategies. Importantly, 17.7% of them corresponded to experimentally validated epitopes. Although this overlap does not constitute experimental validation in the present cohort, it provides independent support for the biological plausibility of the prediction framework and highlights antigenic regions that have already demonstrated immunological relevance in previous studies.

To standardize these findings and facilitate comparisons across studies, Repertoire Size was used to classify HLA alleles according to their predicted antigen presentation capacity. This quantitative framework allows qualitative HLA-binding classifications reported in previous studies to be interpreted in comparable terms [31]. Accordingly, the “strongest” and “weakest” binder categories proposed by Barquera et al. [57] can be interpreted as approximately corresponding to wide (≥1% SB repertoire size) and narrow (<1% candidate epitope repertoire size) repertoires, respectively. This comparison is qualitative, as the underlying thresholds are expected to differ according to the HLA panel, viral dataset, and prediction methodology used.

Previous research has found similar binding affinity profiles across distinct viral families and even with human-derived peptide datasets, with HIV-1 representing the main exception, indicating that peptide-binding affinity profiles may strongly depend on the intrinsic peptide-binding properties of each HLA allele (not necessarily their supertype) and weakly on the viral proteome being evaluated [31,57].

Accordingly, repertoire profiles seen in HLA alleles previously characterized against other viral pathogens generally remained conserved in our HCMV analysis. At the locus level, HLA-A alleles generally exhibited broader predicted peptide repertoires than HLA-B alleles, with HLA-A molecules occupying the upper end of the repertoire-size distribution and HLA-B molecules the lower end in our results (Fig 3), consistent with previous reports [31,57,58].

For instance, the wide-repertoire-size alleles in our dataset, HLA-A*68:01 and the highest-ranking allele HLA-A*02:01, have also displayed broad repertoire sizes for dengue peptides or been classified as Strongest Binders for respiratory viruses or coronaviruses peptides [31,57]. Conversely, HLA-B*15:04 and HLA-B*48:01, alleles showing the narrowest repertoire and no predicted SB across the evaluated HCMV proteins were likewise classified as weakest binders for SARS-CoV-2 [57].

Only A*24:02, B*51:01 and C*04:01, showed intermediate repertoire sizes in our analysis despite having been assigned to narrow-repertoire, weakest-binder, or never-strong-binder categories in previous studies [31,57]. Falling on different categories might be due to the relative thresholds adopted to define categories and the increased stringency of the consensus prediction framework, and, to a lesser extent, the HCMV-specific peptide repertoire. Indeed, the categorization of alleles into Wide-, Intermediate-, and Narrow- Repertoire Size groups was based on the empirical distribution of Repertoire Size values within the study population. Therefore, these classifications represent relative comparisons among the analyzed HLA molecules rather than universal biological categories. Different HLA datasets, pathogens, or prediction frameworks could yield different percentile thresholds. Nevertheless, Repertoire size reflects predicted peptide-HLA binding capacity and should not be interpreted as a direct surrogate of epitope immunogenicity, T-cell magnitude, cytokine production, or clinical protection.

Alleles identified here as wide-repertoire-size alleles, including HLA-A*02:01 and HLA-C*07:02, have previously been associated with HCMV resistance [11] and a superior hierarchy against pp65 and IE1 [13,59]. While C*07:02 resists immunoevasins US2 and US11 [14], its responses may drive age-related immune senescence [60]. Besides, HLA-C locus’ CD8^ + T-cell responses are often limited by lower surface expression compared to HLA-A or -B [13]. Notably, Repertoire Size rankings do not always mirror functional output; for example, A*24:02 elicits significantly stronger IFN-γ responses than A*31:01 despite possessing a narrower Repertoire size [13].

Indeed, while ‘generalist’ alleles (predominantly HLA-A) ensure broad immune visibility by recognizing multiple epitopes, ‘specialist’ alleles (frequently HLA-B) are often associated with superior viral protection despite their narrow repertoire sizes. Their protective efficacy stems from targeting critical, conserved epitopes, as exemplified by HLA-B*57:01 in HIV-1 control [57]. Promiscuity to guarantee presentation, versus specificity to focus the T-cell response, have been initially proposed as distinct revolutionary strategies [61]. However, this specialization is not a pathogen-specific adaptation, as repertoire size hierarchies remain virtually unchanged when evaluated against human self-sequences; hence, it has been stated that presentation breadth is an intrinsic structural property of the HLA molecule rather than an evolutionary response tailored to specific viral proteomes [31].

Regarding the interpretation of the results of this study and their applicability, some limitations should be considered. Although the study cohort comprised kidney transplant candidates and related donors, transplant-related clinical outcomes were not available or analyzed. Therefore, the present findings do not establish associations between HLA variants, predicted HCMV antigen presentation, and transplant-specific outcomes such as HCMV infection, reactivation, disease, graft-related outcomes, or other clinical endpoints. The transplant cohort was used primarily as a source of HLA immunogenetic data, rather than as a clinical cohort for outcome analysis. Consequently, the predicted antigen-presentation profiles should be interpreted as population-level immunogenetic findings that may inform future hypothesis-driven clinical studies, rather than as evidence of transplant-specific clinical associations. Importantly, the present study is entirely based on computational predictions and does not include experimental validation of epitope immunogenicity. Although HLA-peptide binding is a prerequisite for T-cell recognition, predicted binding affinity alone cannot confirm the induction of functional cellular responses. Consequently, the candidate epitopes identified here should be regarded as hypothesis-generating targets that require validation through functional assays such as ELISPOT, intracellular cytokine staining, peptide-MHC multimer analysis, or T-cell proliferation assays. The primary objective of this study was to characterize the predicted antigen-presentation landscape across common HLA alleles rather than to model the complete determinants of immunogenicity. For instance, HLA-peptide affinity alone does not dictate T-cell response; low predicted affinity may still be immunogenic. Moreover, peptide-HLA binding constitutes only one step in the antigen presentation pathway. The generation of an effective T-cell response depends on additional factors [62], including antigen abundance, proteasomal cleavage patterns, TAP-mediated peptide transport, endoplasmic reticulum peptide loading, peptide-HLA complex stability, HLA surface expression levels, immunodominance hierarchies, and the availability and diversity of antigen-specific T-cell receptor repertoires. Consequently, peptides predicted as strong binders may not necessarily induce measurable cellular immune responses, whereas peptides with lower predicted affinity may still be immunogenic *in vivo*. In this context, Repertoire Size represents a relative, prediction-based metric intended to compare antigen recognition potential across common HLA alleles rather than an absolute surrogate of clinical protection. These factors could help explain discrepancies between Repertoire Size rankings and observed *in vitro* or clinical HCMV reactivity. An additional limitation arises from the use of PCR-SSO HLA typing. Although this methodology is routinely employed in transplant histocompatibility laboratories and provides reliable allele-group assignments, it does not achieve full high-resolution typing nor resolve haplotypic phase. This is particularly relevant for HLA-DQ molecules, whose peptide-binding properties depend on the specific pairing of DQA1 and DQB1 chains. Consequently, the HLA-DQ heterodimers could not be appropriately determined; therefore, HLA class II predictions were not included.

In summary, this study provides updated Peruvian HLA data and a characterization of the predicted HCMV antigen-presentation landscape associated with common regional HLA alleles. Though Repertoire Size is not a direct clinical proxy, it defines regional antigen-presentation profiles using global allele data. Identifying common alleles and high-confidence candidate epitopes provides a rational framework for future experimental validation and haplotype-level research [14], and may support the development of HLA-informed immune-monitoring strategies across South American populations. However, these findings should not be interpreted as evidence of transplant-specific risk stratification or clinical associations, which require validation in cohorts with longitudinal clinical and virological outcomes.

## Supporting information

S1 Fig HLA allele group frequencies by locus. All observed HLA allele groups are ranked by prevalence. The dashed line indicates the 5% Allele Frequency (AF) threshold used to define common allele groups within the cohort.
(TIF)

S2 Fig Candidate HLA-I Strong Binders in the 3D structure of HCMV antigens. These figures show regions of experimentally validated epitopes (cyan blue) and novel predicted strong binders (red). Candidate HLA-I strong binders were mapped on the glycoprotein B monomer (A) and trimer (B); this was also done for HLA-II, both in the glycoprotein B monomer (C) and trimer (D). Similarly, on Immediate early protein IE1 monomer (E and G) and homodimer (F and H). Finally, candidate epitopes of HLA class I (I) and class II (J) were mapped on the 65 kDa phosphoprotein monomer.
(TIF)

S1 Table Allele frequencies of all HLA allele groups identified in the cohort. Common allele groups (AF ≥ 5%) in bold. AF, allele frequency.
(XLSX)

S2 Table Reference high-resolution alleles selected by maximum allele frequency (AF). Selection of representative 4-digit HLA alleles for each 2-digit group. The designation was based on the highest reported AF across regional population studies in Peru.
(XLSX)

S3 Table High-resolution HLA allele assignments and estimated minimal frequencies. The 4-digit resolution alleles, their absolute counts and their minimal allele frequency (mAF) are displayed. The mAF was calculated as the ratio of the specific 4-digit allele count to the total number of typed alleles for that locus (including both high- and low-resolution assignments).
(XLSX)

S4 Table Comprehensive list of HCMV-derived HLA epitopes. For each candidate epitope, the source protein, sequence, and coordinates are provided, along with its IEDB status and ID. Structural, functional, and PTM/processing features were cross-referenced with epitope coordinates; overlapping residues for each feature are indicated in brackets. HLA binding is categorized by Total Binding Alleles (all predicted interactions) and SB Alleles (strongly binding interactions only).
(XLSX)

S5 Table HLA-I promiscuous epitopes. Promiscuity is defined as binding ≥6 of 16 HLA-I molecules (35% threshold). Predicted interactions are reported as strong (SB) or weak (WB) binding. Experimentally validated epitopes in bold.
(XLSX)

S1 Material Additional methods for estimation of common HLA alleles. (DOCX)

S1 File Visual abstract: Integrated mapping of HCMV antigenic landscapes. In this manuscript, sequence variability, protein functional features, and predicted HLA-I epitopes were integrated across gB, pp65, and IE1. Entropy analysis identifies variable regions along each antigen, while structural, functional, and post-translational annotations provide protein-level context. Predicted promiscuous epitopes and their overlap with experimentally supported IEDB epitopes highlight regions with broader HLA-I recognition potential. (JPEG)
