# Supplementary material for: HLA-driven variability in human cytomegalovirus immune recognition: Epitope predictions from a kidney transplant Cohort
Source: PLoS One. 2026 Oct 15;21(10):e0360632. doi: 10.1371/journal.pone.0360632 (PMC13671539; doi:10.1371/journal.pone.0360632)
Supplement: Supplementary file 1 [file pone.0360632.s008.docx]

1. **Supplementary Material 1**
   1.1. **Estimation of common HLA alleles**

High-resolution alleles were designated to each common allele group by searching for 4-digit-resolution AFs in Peruvian cohorts both in the literature and in the "Populations by Geographical Regions (Countries)" section of the AFNDB [(1)](https://www.zotero.org/google-docs/?lCHerz). Since AF data of cohorts that we found has been previously reviewed and processed, we selected the allele with the highest AF for each common HLA allele group identified directly from Table S4 in Requena et al. [(2)](https://www.zotero.org/google-docs/?Zp8gX6), except for the C*15 group, whose most frequent allele was found directly in Olvera et al. [(3)](https://www.zotero.org/google-docs/?o29OJB) and the DQA1*03 allele group, for which no 4-digit resolution AFs were available in the literature nor AFNDB, hence we used the most frequent 4-digit resolution HLA allele of this allele group in the studied cohort, which is DQA1*03:01.

1.2. **Epitope annotation and mapping**

Features retrieved from UniProt REST JSON classified into two categories: (i) Structural and Functional features, corresponding to the UniProt feature types: Domain, Motif, Region, Compositional bias, Repeat, Zinc finger, Topological domain, Transmembrane, Disordered, Helix, Strand, Beta strand, Turn, Site, Active site, and Binding sites, and (ii) PTM and Processing features, which included the UniProt feature types: Modified residue (MOD_RES), Glycosylation (CARBOHYD), Lipidation (LIPID), Disulfide bond (DISULFID), Cross-link (CROSSLNK), Signal, Propeptide (PROPEP), Peptide (PEPTIDE), Initiator methionine (INIT_MET), and Chain.

2. **References**

[1](https://www.zotero.org/google-docs/?g3bnxY)**.**	[Gonzalez-Galarza FF, McCabe A, Santos EJMD, Jones J, Takeshita L, Ortega-Rivera ND, et al. Allele frequency net database (AFND) 2020 update: gold-standard data classification, open access genotype data and new query tools. Nucleic Acids Res. 2019 Nov 13;gkz1029.](https://www.zotero.org/google-docs/?g3bnxY)

[2.](https://www.zotero.org/google-docs/?g3bnxY)	[Requena D, Médico A, Chacón RD, Ramírez M, Marín-Sánchez O. Identification of Novel Candidate Epitopes on SARS-CoV-2 Proteins for South America: A Review of HLA Frequencies by Country. Front Immunol. 2020;11:2008.](https://www.zotero.org/google-docs/?g3bnxY)

[3.](https://www.zotero.org/google-docs/?g3bnxY)	[Olvera A, Pérez-Álvarez S, Ibarrondo J, Ganoza C, Lama JR, Lucchetti A, et al. The HLA-C*04: 01/KIR2DS4 gene combination and human leukocyte antigen alleles with high population frequency drive rate of HIV disease progression. AIDS. 2015 Mar 13;29(5):507–17.](https://www.zotero.org/google-docs/?g3bnxY)
