# Supplementary material for: HLA-driven variability in human cytomegalovirus immune recognition: Epitope predictions from a kidney transplant Cohort
Source: PLoS One. 2026 Oct 15;21(10):e0360632. doi: 10.1371/journal.pone.0360632 (PMC13671539; doi:10.1371/journal.pone.0360632)
Supplement: Supplementary file 2 [file pone.0360632.s011.docx]

**Journal Requirements:**

**Comment J.1.**

When submitting your revision, we need you to address these additional requirements.

1. Please ensure that your manuscript meets PLOS ONE's style requirements, including those for file naming. The PLOS ONE style templates can be found at

[https://journals.plos.org/plosone/s/file?id=wjVg/PLOSOne_formatting_sample_main_body.pdf](https://track.editorialmanager.com/CL0/https:%2F%2Fjournals.plos.org%2Fplosone%2Fs%2Ffile%3Fid=wjVg%2FPLOSOne_formatting_sample_main_body.pdf/1/010f019e72dd39dc-09124258-6aa6-4ee6-8d75-44f091b9ff1d-000000/CPF-olT7b1WNPrwGwkqx6hSAJTkyMSvCyBpDAFJm-aY=258) and

[https://journals.plos.org/plosone/s/file?id=ba62/PLOSOne_formatting_sample_title_authors_affiliations.pdf](https://track.editorialmanager.com/CL0/https:%2F%2Fjournals.plos.org%2Fplosone%2Fs%2Ffile%3Fid=ba62%2FPLOSOne_formatting_sample_title_authors_affiliations.pdf/1/010f019e72dd39dc-09124258-6aa6-4ee6-8d75-44f091b9ff1d-000000/7tHVsjcLYkTkL9QB5LTkwNPzUev4qPPcAH7__JO8gTQ=258)

**Response J.1.**

Thank you for these comments. In this version, we adhere to the journal guidelines in terms of style and formatting.

**Comment J.2.**

2. Please ensure that you refer to Figure 2 in your text as, if accepted, production will need this reference to link the reader to the figure.

**Response J.2.**

Thank you for pointing this out. After revising the flow and organization of the manuscript, we considered that this figure is better suited as supplementary material. Therefore, it has been moved to the Supplementary Information and is now presented as Figure S2 (S2 Fig). We have also included the corresponding reference to Figure S2 in the main text where appropriate.

**Comment J.3.**

3. Please include captions for your Supporting Information files at the end of your manuscript, and update any in-text citations to match accordingly. Please see our Supporting Information guidelines for more information: [http://journals.plos.org/plosone/s/supporting-information](http://track.editorialmanager.com/CL0/http:%2F%2Fjournals.plos.org%2Fplosone%2Fs%2Fsupporting-information/1/010f019e72dd39dc-09124258-6aa6-4ee6-8d75-44f091b9ff1d-000000/5KRJrjUWdsK5c0LXjRZ72zGsHVC7I7VUil8nAvQU5pg=258).

**Response J.3.**

Thank you for pointing this out. In this version, we followed the Journal format style and included in-text citations for supporting information.

**Comment J.4.**

4. If the reviewer comments include a recommendation to cite specific previously published works, please review and evaluate these publications to determine whether they are relevant and should be cited. There is no requirement to cite these works unless the editor has indicated otherwise.
**Response J.4.**

Thank you for this comment. We have revised all reviewers' and editors' comments carefully.

**Comment J.5.**
5. Please review your reference list to ensure that it is complete and correct. If you have cited papers that have been retracted, please include the rationale for doing so in the manuscript text, or remove these references and replace them with relevant current references. Any changes to the reference list should be mentioned in the rebuttal letter that accompanies your revised manuscript. If you need to cite a retracted article, indicate the article’s retracted status in the References list and also include a citation and full reference for the retraction notice.

**Response J.5.**

Thank you for this comment. We have revised all references and in-text citations carefully.

**Editor Comments:**

**Additional Editor Comments:**

Minor revision.

**Response:**

Thank you for the decision on our manuscript. We have revised all reviewers' comments carefully.

**Reviewers Comments:**

**Reviewer #1:**

**Comment 1.1.**

The manuscript is well written with adequate analysis done and data well presented. I however advise to add a Demographic table as part of the results to show the study population data, donors, recipients, age, males vs females those on dialysis and so on. Overall this is a good study and gives inside into HMCV and the role of HLA in immune response.

**Response 1.1:**

Thank you for this valuable suggestion. Following your recommendation, we have added a new subsection entitled "Study Cohort" to the Results section, together with a demographic table summarizing the characteristics of the study population. This table includes the number of transplant candidates (recipients) and family members evaluated as potential living donors, age distribution, sex distribution, and the relationship of the evaluated donors to the transplant candidates. We believe these additions provide a clearer description of the study population and improve the interpretation of the subsequent analyses.

Regarding dialysis status, all individuals included in the Case group corresponded to kidney transplant candidates undergoing pre-transplant evaluation, whereas the Family group consisted of their related potential living donors.

**Reviewer #2:**

**Comment 2.1.**
This manuscript presents an analysis of HLA allele distributions and predicted HCMV-specific T-cell epitopes in a Peruvian transplant cohort using in silico approaches. The study addresses an underrepresented population and provides potentially valuable regional HLA data. However, the current version relies heavily on computational predictions without sufficient biological or clinical validation, which limits its impact and interpretability.

Therefore, substantial revisions are required before this manuscript can be considered for publication.
**Response 2.1:**

Thank you for your positive evaluation of our manuscript. We have thoroughly revised the manuscript in response to all reviewers’ comments and suggestions, and we trust that the revised version satisfactorily addresses the concerns raised and meets the journal’s publication criteria.

**Comment 2.2.**
This study relies entirely on in silico epitope prediction without functional validation (e.g., ELISPOT or intracellular cytokine assays). As a result, the actual immunogenicity of the predicted epitopes remains uncertain. The conclusions regarding immune responses should be interpreted with caution, and the limitations should be more clearly emphasized.
**Response 2.2:**

We thank the reviewer for this important observation. We agree that computational prediction of HLA-binding epitopes does not constitute direct evidence of immunogenicity and that functional validation is required to confirm T-cell responses. Accordingly, we revised the Abstract, Introduction, Discussion, and Conclusions to consistently describe our findings as predictions of antigen-presentation potential rather than demonstrated immune responses. We also clarified throughout the manuscript that HLA-peptide binding represents only one component of the antigen presentation pathway and that experimental validation remains necessary to confirm the biological relevance of the identified epitopes.

In addition, we expanded the comparison with experimentally validated HCMV epitopes available in IEDB and incorporated a new figure showing the distribution of validated and newly predicted epitopes across the analyzed proteins. A proportion of the predicted candidates corresponded to previously reported IEDB epitopes, supporting the biological plausibility of the prediction framework. While these overlaps do not constitute experimental validation in our cohort, they help prioritize candidate epitopes for future functional studies. The changes are highlighted in yellow.

**Comment 2.3.**
Although a transplant cohort was included, no clinical data were analyzed. This limits the clinical applicability of the findings. The rationale for using a transplant population should be better justified or supported with clinical correlations.
**Response 2.3:**

We thank the reviewer for this valuable comment. We agree that, in the absence of clinical outcome analyses, the rationale for selecting a kidney transplant cohort should be better justified. Accordingly, we expanded the Introduction to clarify that kidney transplant candidates and their related donors provide a valuable source of immunogenetic data because HLA typing is routinely performed as part of donor–recipient matching. In addition, we incorporated a new Study Cohort subsection and a demographic table to better describe the study population. We believe these modifications better justify the selected cohort while maintaining the focus of the study on population immunogenetics rather than clinical associations.

**Comment 2.4.**
PCR-SSO provides only intermediate resolution and does not allow phasing of HLA alleles. This is particularly critical for HLA-DQ, where α/β pairing determines epitope binding. This limitation may affect the reliability of HLA class II predictions and should be discussed in more detail.

**Response 2.4:**

We thank the reviewer for highlighting this important limitation. Because PCR-SSO typing does not provide phased HLA-DQ haplotypes, uncertainty exists regarding the exact DQA1/DQB1 pairings present in individual subjects. We agree that this limitation may affect the reliability of HLA class II binding predictions.

To address this concern and improve methodological robustness, we refocused the study on HLA class I analyses, which are not affected by DQ pairing ambiguity and are directly relevant to antiviral CD8+ T-cell responses against HCMV. Consequently, HLA class II epitope prediction analyses were removed from the revised manuscript. The changes are highlighted in yellow.

**Comment 2.5.**
The study focuses on HLA-peptide binding affinity but does not consider key determinants of immunogenicity, such as antigen processing, HLA surface expression, and TCR repertoire. Binding predictions alone are insufficient to infer actual immune responses.

**Response 2.5:**

We thank the reviewer for this important comment. We agree that peptide-HLA binding represents only one component of the complex process leading to T-cell activation and immunogenicity. Additional factors, including antigen processing, peptide loading, HLA expression, immunodominance, and T-cell receptor (TCR) repertoire diversity, can substantially influence immune responses.

Our objective was not to predict immunogenicity directly, but rather to characterize the predicted antigen-presentation landscape associated with common HLA variants in a Peruvian cohort, which is now declared in the aims of the study. Accordingly, we expanded the Discussion section and revised the manuscript to consistently interpret our findings as estimates of peptide-presentation potential rather than direct measures of T-cell responsiveness, cytokine production, or clinical protection. We also emphasize that PSBD reflects predicted HLA-binding capacity and that experimental validation will be required to determine the biological relevance of the identified epitopes. The changes are highlighted in yellow.

**Comment 2.6.**
The definition of PSBD may introduce bias due to differences in protein length and the use of arbitrary percentile thresholds (P25/P75).
The rationale for these thresholds is not clearly justified. Additional clarification is required.

**Response 2.6:**

Thank you for this important observation. To minimize bias related to protein size, the Predicted Strong Binder Density (PSBD) was calculated as the number of predicted strong binders normalized to the length of the source protein, allowing standardized comparisons across proteins of different sizes. We also agree that repertoire size categories should not rely on arbitrary thresholds. Accordingly, we revised the methodology and established repertoire size groups using the Jenks natural breaks method, which identifies natural groupings within the observed data distribution. We further clarified that these categories represent relative rankings within the analyzed dataset and should not be interpreted as biologically validated thresholds. The changes are highlighted in yellow.

**Comment 2.7.**
The structural mapping is largely descriptive and lacks functional interpretation. In particular, the use of a modeled pp65 structure raises concerns regarding accuracy. The biological relevance of the structural mapping should be better explained. Figure 2 is visually detailed but its scientific message is unclear. It does not provide additional mechanistic or functional insight beyond the prediction results and may benefit from simplification and clearer interpretation.

**Response 2.7:**

Thank you for this insightful comment. We agree that the structural mapping analysis is primarily descriptive and that its biological interpretation is limited, particularly for pp65, whose structure was generated using computational modeling due to the absence of a complete experimentally resolved structure.

In response to this concern, we revised the manuscript to reduce the emphasis placed on structural mapping and moved the corresponding figure from the main text to the Supplementary Material (now Figure S2). We also clarified that the purpose of this analysis is to provide a qualitative visualization of the spatial distribution of known and predicted epitopes and to support future experimental prioritization, rather than to infer functional consequences or mechanisms of immune recognition.

In addition, to improve the functional interpretation of the epitope predictions, we incorporated a new figure showing the linear distribution of experimentally validated (IEDB) and newly predicted epitopes across the analyzed HCMV proteins. This representation integrates epitope location with annotated protein features, including functional domains, post-translational modification sites, and sequence conservation patterns derived from comparative sequence analyses. The changes are highlighted in yellow.
