# Supplementary material for: HLA-driven variability in human cytomegalovirus immune recognition: Epitope predictions from a kidney transplant Cohort
Source: PLoS One. 2026 Oct 15;21(10):e0360632. doi: 10.1371/journal.pone.0360632 (PMC13671539; doi:10.1371/journal.pone.0360632)
Supplement: Supplementary file 3 [file pone.0360632.s012.docx]

**Journal Requirements:**

Comment J.1.

If the reviewer comments include a recommendation to cite specific previously published works, please review and evaluate these publications to determine whether they are relevant and should be cited. There is no requirement to cite these works unless the editor has indicated otherwise.

Please review your reference list to ensure that it is complete and correct. If you have cited papers that have been retracted, please include the rationale for doing so in the manuscript text, or remove these references and replace them with relevant current references. Any changes to the reference list should be mentioned in the rebuttal letter that accompanies your revised manuscript. If you need to cite a retracted article, indicate the article’s retracted status in the References list and also include a citation and full reference for the retraction notice.

**Response J.1**

We thank the Editorial Office for these important recommendations regarding the verification of the reference list. In response, we manually reviewed the references cited in the revised manuscript and checked their publication status and bibliographic information.

During this review, we identified two references associated with a published corrigendum/erratum. We carefully examined the corresponding correction notices and confirmed that the reported corrections concern minor details and do not affect the findings, interpretation, or specific information for which these articles were cited in our manuscript. Therefore, we consider these references to remain relevant and appropriate for the purposes for which they are cited.

For transparency, the DOI of the corresponding corrigendum/erratum notices will be provided below:

1. Reference [9] – Al Naqbi H, Mawart A, Alshamsi J, Al Safar H, Tay GK. Major histocompatibility complex (MHC) associations with diseases in ethnic groups of the Arabian Peninsula. Immunogenetics. 2021;73: 131–152. doi:10.1007/s00251-021-01204-x
2. Reference [28] – O’Donnell TJ, Rubinsteyn A, Laserson U. MHCflurry 2.0: Improved Pan-Allele Prediction of MHC Class I-Presented Peptides by Incorporating Antigen Processing. Cell Syst. 2020;11: 42-48.e7. doi:10.1016/j.cels.2020.06.010

Neither of these articles has been identified as retracted. Accordingly, no references were removed on the basis of retraction. We have retained the two references because the published corrections do not alter the scientific rationale or conclusions supporting their citation in the present manuscript.

**Reviewer #1:**

**Comment 1.1.**

Reviewe 1 had requested a demographics table to be added, Although the authours addressed the comment with a statement and not a in a table format. I accept that they have satisfied the reviewer's comment.

**Response 1.1.**

We thank the reviewer for confirming that the addition of the demographic information adequately addresses the previous comment. We appreciate the reviewer’s consideration of this revision and are pleased that the information provided in the revised manuscript was considered sufficient to address the concern.

**Reviewer #2:**

**Comment 2.1.**

I have reviewed the revised manuscript, and the authors have adequately addressed most of my previous comments. The manuscript has been substantially improved. However, an additional clarification is needed regarding Comment 2.3.

The rationale for selecting the kidney transplant cohort has been clarified. However, the Discussion still does not explicitly acknowledge the absence of transplant-related clinical outcome analyses. Please add this as a limitation and clarify that the present findings do not establish transplant-specific clinical associations. In this context, the statement regarding “risk stratification” in the Conclusion should also be tempered accordingly.

**Response 2.1.**

We thank the reviewer for this important clarification. We agree that, although the rationale for selecting the kidney transplant cohort has now been clarified, the manuscript should explicitly acknowledge that transplant-related clinical outcomes were not analyzed. We have therefore added a specific statement in the Discussion identifying the absence of transplant-related clinical and virological outcome analyses as a limitation. We now clarify that the transplant cohort was used primarily as a source of HLA immunogenetic data and that the present findings do not establish associations between HLA variants, predicted HCMV antigen presentation, and transplant-specific clinical outcomes.

In addition, we have tempered the wording regarding “risk stratification” in the Conclusion and clarified that the predicted antigen-presentation profiles may inform future HLA-informed immune-monitoring studies, but should not be interpreted as evidence of transplant-specific risk stratification or clinical associations. We have also aligned the Abstract Conclusion with this clarification to ensure consistency throughout the manuscript.
