# Supplementary material for: HLA-driven variability in human cytomegalovirus immune recognition: Epitope predictions from a kidney transplant Cohort
Source: PLoS One. 2026 Oct 15;21(10):e0360632. doi: 10.1371/journal.pone.0360632 (PMC13671539; doi:10.1371/journal.pone.0360632)
Supplement: Peer review history

# Author comment: Author response to Decision Letter 0

**DOI:** 10.1371/journal.pone.0360632.r001

**Transfer Alert**

This paper was transferred from another journal. As a result, its full editorial history (including decision letters, peer reviews and author responses) may not be present.

28 Feb 2026

# Peer review: Decision Letter 0

**DOI:** 10.1371/journal.pone.0360632.r002

**Authors:** Maemu Gededzha

29 May 2026

Dear Dr. Carrasco,

Thank you for submitting your manuscript to PLOS ONE. After careful consideration, we feel that it has merit but does not fully meet PLOS ONE’s publication criteria as it currently stands. Therefore, we invite you to submit a revised version of the manuscript that addresses the points raised during the review process.

plosone@plos.org. When you're ready to submit your revision, log on to https://www.editorialmanager.com/pone/ and select the 'Submissions Needing Revision' folder to locate your manuscript file.

- A letter that responds to each point raised by the academic editor and reviewer(s). You should upload this letter as a separate file labeled 'Response to Reviewers'.
- A marked-up copy of your manuscript that highlights changes made to the original version. You should upload this as a separate file labeled 'Revised Manuscript with Track Changes'.
- An unmarked version of your revised paper without tracked changes. You should upload this as a separate file labeled 'Manuscript'.

If you would like to make changes to your financial disclosure, please include your updated statement in your cover letter. Guidelines for resubmitting your figure files are available below the reviewer comments at the end of this letter.

If applicable, we recommend that you deposit your laboratory protocols in protocols.io to enhance the reproducibility of your results. Protocols.io assigns your protocol its own identifier (DOI) so that it can be cited independently in the future. For instructions see: https://journals.plos.org/plosone/s/submission-guidelines#loc-laboratory-protocols. Additionally, PLOS ONE offers an option for publishing peer-reviewed Lab Protocol articles, which describe protocols hosted on protocols.io. Read more information on sharing protocols at https://plos.org/protocols?utm_medium=editorial-email&utm_source=authorletters&utm_campaign=protocols.

As the corresponding author, your ORCID iD is verified in the submission system and will appear in the published article. PLOS supports the use of ORCID, and we encourage all coauthors to register for an ORCID iD and use it as well. Please encourage your coauthors to verify their ORCID iD within the submission system before final acceptance, as unverified ORCID iDs will not appear in the published article. *Only*  the individual author can complete the verification step; PLOS staff *cannot*  verify ORCID iDs on behalf of authors.

We look forward to receiving your revised manuscript.

Kind regards,

Maemu Petronella Gededzha, Ph.D

Academic Editor

PLOS One

**Journal Requirements:**

When submitting your revision, we need you to address these additional requirements.

1. Please ensure that your manuscript meets PLOS ONE's style requirements, including those for file naming. The PLOS ONE style templates can be found at

https://journals.plos.org/plosone/s/file?id=wjVg/PLOSOne_formatting_sample_main_body.pdf and

https://journals.plos.org/plosone/s/file?id=ba62/PLOSOne_formatting_sample_title_authors_affiliations.pdf

2. Please ensure that you refer to Figure 2 in your text as, if accepted, production will need this reference to link the reader to the figure.

3. Please include captions for your Supporting Information files at the end of your manuscript, and update any in-text citations to match accordingly. Please see our Supporting Information guidelines for more information: http://journals.plos.org/plosone/s/supporting-information.

4. If the reviewer comments include a recommendation to cite specific previously published works, please review and evaluate these publications to determine whether they are relevant and should be cited. There is no requirement to cite these works unless the editor has indicated otherwise.

5. Please review your reference list to ensure that it is complete and correct. If you have cited papers that have been retracted, please include the rationale for doing so in the manuscript text, or remove these references and replace them with relevant current references. Any changes to the reference list should be mentioned in the rebuttal letter that accompanies your revised manuscript. If you need to cite a retracted article, indicate the article’s retracted status in the References list and also include a citation and full reference for the retraction notice.

**Additional Editor Comments:**

Minor revision.

[Note: HTML markup is below. Please do not edit.]

Reviewers' comments:

Reviewer's Responses to Questions

**Comments to the Author**

1. Is the manuscript technically sound, and do the data support the conclusions?

Reviewer #1: Yes

Reviewer #2: Yes

**********

2. Has the statistical analysis been performed appropriately and rigorously?

Reviewer #1: Yes

Reviewer #2: I Don't Know

**********

3. Have the authors made all data underlying the findings in their manuscript fully available?

The PLOS Data policy

Reviewer #1: Yes

Reviewer #2: Yes

**********

4. Is the manuscript presented in an intelligible fashion and written in standard English?

Reviewer #1: Yes

Reviewer #2: Yes

**********

Reviewer #1: The manuscript is well written with adequate analysis done and data well presented. I however advise to add a Demographic table as part of the results to show the study population data, donors, recipients, age, malesvs females those on dialysis and so on. Overall this is a good study and gives inside into HMCV and the role of HLA in immune response.

Reviewer #2: This manuscript presents an analysis of HLA allele distributions and predicted HCMV-specific T-cell epitopes in a Peruvian transplant cohort using in silico approaches. The study addresses an underrepresented population and provides potentially valuable regional HLA data. However, the current version relies heavily on computational predictions without sufficient biological or clinical validation, which limits its impact and interpretability.

Therefore, substantial revisions are required before this manuscript can be considered for publication.

This study relies entirely on in silico epitope prediction without functional validation (e.g., ELISPOT or intracellular cytokine assays). As a result, the actual immunogenicity of the predicted epitopes remains uncertain. The conclusions regarding immune responses should be interpreted with caution, and the limitations should be more clearly emphasized.

Although a transplant cohort was included, no clinical data were analyzed. This limits the clinical applicability of the findings. The rationale for using a transplant population should be better justified or supported with clinical correlations.

PCR-SSO provides only intermediate resolution and does not allow phasing of HLA alleles. This is particularly critical for HLA-DQ, where α/β pairing determines epitope binding. This limitation may affect the reliability of HLA class II predictions and should be discussed in more detail.

The study focuses on HLA-peptide binding affinity but does not consider key determinants of immunogenicity, such as antigen processing, HLA surface expression, and TCR repertoire. Binding predictions alone are insufficient to infer actual immune responses.

The definition of PSBD may introduce bias due to differences in protein length and the use of arbitrary percentile thresholds (P25/P75).

The rationale for these thresholds is not clearly justified. Additional clarification is required.

The structural mapping is largely descriptive and lacks functional interpretation. In particular, the use of a modeled pp65 structure raises concerns regarding accuracy. The biological relevance of the structural mapping should be better explained.

Figure 2 is visually detailed but its scientific message is unclear. It does not provide additional mechanistic or functional insight beyond the prediction results and may benefit from simplification and clearer interpretation.

**********

what does this mean?). If published, this will include your full peer review and any attached files.

If you choose “no”, your identity will remain anonymous but your review may still be made public.

**Do you want your identity to be public for this peer review?**  For information about this choice, including consent withdrawal, please see our Privacy Policy

Reviewer #1: No

Reviewer #2: No

**********

[NOTE: If reviewer comments were submitted as an attachment file, they will be attached to this email and accessible via the submission site. Please log into your account, locate the manuscript record, and check for the action link "View Attachments". If this link does not appear, there are no attachment files.]

To ensure your figures meet our technical requirements, please review our figure guidelines: https://journals.plos.org/plosone/s/figures

You may also use PLOS’s free figure tool, NAAS, to help you prepare publication quality figures: https://journals.plos.org/plosone/s/figures#loc-tools-for-figure-preparation.

NAAS will assess whether your figures meet our technical requirements by comparing each figure against our figure specifications.

# Author comment: Author response to Decision Letter 1

**DOI:** 10.1371/journal.pone.0360632.r003

29 Jul 2026

Journal Requirements:

Comment J.1.

When submitting your revision, we need you to address these additional requirements.

1. Please ensure that your manuscript meets PLOS ONE's style requirements, including those for file naming. The PLOS ONE style templates can be found at

https://journals.plos.org/plosone/s/file?id=wjVg/PLOSOne_formatting_sample_main_body.pdf and

https://journals.plos.org/plosone/s/file?id=ba62/PLOSOne_formatting_sample_title_authors_affiliations.pdf

Response J.1.

Thank you for these comments. In this version, we adhere to the journal guidelines in terms of style and formatting.

Comment J.2.

2. Please ensure that you refer to Figure 2 in your text as, if accepted, production will need this reference to link the reader to the figure.

Response J.2.

Thank you for pointing this out. After revising the flow and organization of the manuscript, we considered that this figure is better suited as supplementary material. Therefore, it has been moved to the Supplementary Information and is now presented as Figure S2 (S2 Fig). We have also included the corresponding reference to Figure S2 in the main text where appropriate.

Comment J.3.

3. Please include captions for your Supporting Information files at the end of your manuscript, and update any in-text citations to match accordingly. Please see our Supporting Information guidelines for more information: http://journals.plos.org/plosone/s/supporting-information.

Response J.3.

Thank you for pointing this out. In this version, we followed the Journal format style and included in-text citations for supporting information.

Comment J.4.

4. If the reviewer comments include a recommendation to cite specific previously published works, please review and evaluate these publications to determine whether they are relevant and should be cited. There is no requirement to cite these works unless the editor has indicated otherwise.

Response J.4.

Thank you for this comment. We have revised all reviewers' and editors' comments carefully.

Comment J.5.

5. Please review your reference list to ensure that it is complete and correct. If you have cited papers that have been retracted, please include the rationale for doing so in the manuscript text, or remove these references and replace them with relevant current references. Any changes to the reference list should be mentioned in the rebuttal letter that accompanies your revised manuscript. If you need to cite a retracted article, indicate the article’s retracted status in the References list and also include a citation and full reference for the retraction notice.

Response J.5.

Thank you for this comment. We have revised all references and in-text citations carefully.

Editor Comments:

Additional Editor Comments:

Minor revision.

Response:

Thank you for the decision on our manuscript. We have revised all reviewers' comments carefully.

Reviewers Comments:

Reviewer #1:

Comment 1.1.

The manuscript is well written with adequate analysis done and data well presented. I however advise to add a Demographic table as part of the results to show the study population data, donors, recipients, age, males vs females those on dialysis and so on. Overall this is a good study and gives inside into HMCV and the role of HLA in immune response.

Response 1.1:

Thank you for this valuable suggestion. Following your recommendation, we have added a new subsection entitled "Study Cohort" to the Results section, together with a demographic table summarizing the characteristics of the study population. This table includes the number of transplant candidates (recipients) and family members evaluated as potential living donors, age distribution, sex distribution, and the relationship of the evaluated donors to the transplant candidates. We believe these additions provide a clearer description of the study population and improve the interpretation of the subsequent analyses.

Regarding dialysis status, all individuals included in the Case group corresponded to kidney transplant candidates undergoing pre-transplant evaluation, whereas the Family group consisted of their related potential living donors.

Reviewer #2:

Comment 2.1.

This manuscript presents an analysis of HLA allele distributions and predicted HCMV-specific T-cell epitopes in a Peruvian transplant cohort using in silico approaches. The study addresses an underrepresented population and provides potentially valuable regional HLA data. However, the current version relies heavily on computational predictions without sufficient biological or clinical validation, which limits its impact and interpretability.

Therefore, substantial revisions are required before this manuscript can be considered for publication.

Response 2.1:

Thank you for your positive evaluation of our manuscript. We have thoroughly revised the manuscript in response to all reviewers’ comments and suggestions, and we trust that the revised version satisfactorily addresses the concerns raised and meets the journal’s publication criteria.

Comment 2.2.

This study relies entirely on in silico epitope prediction without functional validation (e.g., ELISPOT or intracellular cytokine assays). As a result, the actual immunogenicity of the predicted epitopes remains uncertain. The conclusions regarding immune responses should be interpreted with caution, and the limitations should be more clearly emphasized.

Response 2.2:

We thank the reviewer for this important observation. We agree that computational prediction of HLA-binding epitopes does not constitute direct evidence of immunogenicity and that functional validation is required to confirm T-cell responses. Accordingly, we revised the Abstract, Introduction, Discussion, and Conclusions to consistently describe our findings as predictions of antigen-presentation potential rather than demonstrated immune responses. We also clarified throughout the manuscript that HLA-peptide binding represents only one component of the antigen presentation pathway and that experimental validation remains necessary to confirm the biological relevance of the identified epitopes.

In addition, we expanded the comparison with experimentally validated HCMV epitopes available in IEDB and incorporated a new figure showing the distribution of validated and newly predicted epitopes across the analyzed proteins. A proportion of the predicted candidates corresponded to previously reported IEDB epitopes, supporting the biological plausibility of the prediction framework. While these overlaps do not constitute experimental validation in our cohort, they help prioritize candidate epitopes for future functional studies. The changes are highlighted in yellow.

Comment 2.3.

Although a transplant cohort was included, no clinical data were analyzed. This limits the clinical applicability of the findings. The rationale for using a transplant population should be better justified or supported with clinical correlations.

Response 2.3:

We thank the reviewer for this valuable comment. We agree that, in the absence of clinical outcome analyses, the rationale for selecting a kidney transplant cohort should be better justified. Accordingly, we expanded the Introduction to clarify that kidney transplant candidates and their related donors provide a valuable source of immunogenetic data because HLA typing is routinely performed as part of donor–recipient matching. In addition, we incorporated a new Study Cohort subsection and a demographic table to better describe the study population. We believe these modifications better justify the selected cohort while maintaining the focus of the study on population immunogenetics rather than clinical associations.

Comment 2.4.

PCR-SSO provides only intermediate resolution and does not allow phasing of HLA alleles. This is particularly critical for HLA-DQ, where α/β pairing determines epitope binding. This limitation may affect the reliability of HLA class II predictions and should be discussed in more detail.

Response 2.4:

We thank the reviewer for highlighting this important limitation. Because PCR-SSO typing does not provide phased HLA-DQ haplotypes, uncertainty exists regarding the exact DQA1/DQB1 pairings present in individual subjects. We agree that this limitation may affect the reliability of HLA class II binding predictions.

To address this concern and improve methodological robustness, we refocused the study on HLA class I analyses, which are not affected by DQ pairing ambiguity and are directly relevant to antiviral CD8+ T-cell responses against HCMV. Consequently, HLA class II epitope prediction analyses were removed from the revised manuscript. The changes are highlighted in yellow.

Comment 2.5.

The study focuses on HLA-peptide binding affinity but does not consider key determinants of immunogenicity, such as antigen processing, HLA surface expression, and TCR repertoire. Binding predictions alone are insufficient to infer actual immune responses.

Response 2.5:

We thank the reviewer for this important comment. We agree that peptide-HLA binding represents only one component of the complex process leading to T-cell activation and immunogenicity. Additional factors, including antigen processing, peptide loading, HLA expression, immunodominance, and T-cell receptor (TCR) repertoire diversity, can substantially influence immune responses.

Our objective was not to predict immunogenicity directly, but rather to characterize the predicted antigen-presentation landscape associated with common HLA variants in a Peruvian cohort, which is now declared in the aims of the study. Accordingly, we expanded the Discussion section and revised the manuscript to consistently interpret our findings as estimates of peptide-presentation potential rather than direct measures of T-cell responsiveness, cytokine production, or clinical protection. We also emphasize that PSBD reflects predicted HLA-binding capacity and that experimental validation will be required to determine the biological relevance of the identified epitopes. The changes are highlighted in yellow.

Comment 2.6.

The definition of PSBD may introduce bias due to differences in protein length and the use of arbitrary percentile thresholds (P25/P75).

The rationale for these thresholds is not clearly justified. Additional clarification is required.

Response 2.6:

Thank you for this important observation. To minimize bias related to protein size, the Predicted Strong Binder Density (PSBD) was calculated as the number of predicted strong binders normalized to the length of the source protein, allowing standardized comparisons across proteins of different sizes. We also agree that repertoire size categories should not rely on arbitrary thresholds. Accordingly, we revised the methodology and established repertoire size groups using the Jenks natural breaks method, which identifies natural groupings within the observed data distribution. We further clarified that these categories represent relative rankings within the analyzed dataset and should not be interpreted as biologically validated thresholds. The changes are highlighted in yellow.

Comment 2.7.

The structural mapping is largely descriptive and lacks functional interpretation. In particular, the use of a modeled pp65 structure raises concerns regarding accuracy. The biological relevance of the structural mapping should be better explained. Figure 2 is visually detailed but its scientific message is unclear. It does not provide additional mechanistic or functional insight beyond the prediction results and may benefit from simplification and clearer interpretation.

Response 2.7:

Thank you for this insightful comment. We agree that the structural mapping analysis is primarily descriptive and that its biological interpretation is limited, particularly for pp65, whose structure was generated using computational modeling due to the absence of a complete experimentally resolved structure.

In response to this concern, we revised the manuscript to reduce the emphasis placed on structural mapping and moved the corresponding figure from the main text to the Supplementary Material (now Figure S2). We also clarified that the purpose of this analysis is to provide a qualitative visualization of the spatial distribution of known and predicted epitopes and to support future experimental prioritization, rather than to infer functional consequences or mechanisms of immune recognition.

In addition, to improve the functional interpretation of the epitope predictions, we incorporated a new figure showing the linear distribution of experimentally validated (IEDB) and newly predicted epitopes across the analyzed HCMV proteins. This representation integrates epitope location with annotated protein features, including functional domains, post-translational modification sites, and sequence conservation patterns derived from comparative sequence analyses. The changes are highlighted in yellow.

Attachment Submitted filename: Letter to Journal_Editor_Reviewers.docx

# Peer review: Decision Letter 1

**DOI:** 10.1371/journal.pone.0360632.r004

**Authors:** Maemu Gededzha

26 Aug 2026

Thank you for submitting your manuscript to PLOS One. After careful consideration, we feel that it has merit but does not fully meet PLOS One’s publication criteria as it currently stands. Therefore, we invite you to submit a revised version of the manuscript that addresses the points raised during the review process.

Please submit your revised manuscript by Oct 10 2026 11:59PM. If you will need more time than this to complete your revisions, please reply to this message or contact the journal office at plosone@plos.org. When you're ready to submit your revision, log on to https://www.editorialmanager.com/pone/ and select the 'Submissions Needing Revision' folder to locate your manuscript file.

- A letter that responds to each point raised by the academic editor and reviewer(s). You should upload this letter as a separate file labeled 'Response to Reviewers'.
- A marked-up copy of your manuscript that highlights changes made to the original version. You should upload this as a separate file labeled 'Revised Manuscript with Track Changes'.
- An unmarked version of your revised paper without tracked changes. You should upload this as a separate file labeled 'Manuscript'.

If you would like to make changes to your financial disclosure, please include your updated statement in your cover letter. Guidelines for resubmitting your figure files are available below the reviewer comments at the end of this letter.

If applicable, we recommend that you deposit your laboratory protocols in protocols.io to enhance the reproducibility of your results. Protocols.io assigns your protocol its own identifier (DOI) so that it can be cited independently in the future. For instructions see: https://journals.plos.org/plosone/s/submission-guidelines#loc-laboratory-protocols. Additionally, PLOS One offers an option for publishing peer-reviewed Lab Protocol articles, which describe protocols hosted on protocols.io. Read more information on sharing protocols at https://plos.org/protocols?utm_medium=editorial-email&utm_source=authorletters&utm_campaign=protocols.

As the corresponding author, your ORCID iD is verified in the submission system and will appear in the published article. PLOS supports the use of ORCID, and we encourage all coauthors to register for an ORCID iD and use it as well. Please encourage your coauthors to verify their ORCID iD within the submission system before final acceptance, as unverified ORCID iDs will not appear in the published article. *Only*  the individual author can complete the verification step; PLOS staff *cannot*  verify ORCID iDs on behalf of authors.

We look forward to receiving your revised manuscript.

Kind regards,

Maemu Petronella Gededzha, Ph.D

Academic Editor

PLOS One

Journal Requirements:

If the reviewer comments include a recommendation to cite specific previously published works, please review and evaluate these publications to determine whether they are relevant and should be cited. There is no requirement to cite these works unless the editor has indicated otherwise.

Please review your reference list to ensure that it is complete and correct. If you have cited papers that have been retracted, please include the rationale for doing so in the manuscript text, or remove these references and replace them with relevant current references. Any changes to the reference list should be mentioned in the rebuttal letter that accompanies your revised manuscript. If you need to cite a retracted article, indicate the article’s retracted status in the References list and also include a citation and full reference for the retraction notice.

Additional Editor Comments:

Please address the outstanding comments as per reviewers comments

[Note: HTML markup is below. Please do not edit.]

Reviewers' comments:

Reviewer's Responses to Questions

**Comments to the Author**

Reviewer #1: All comments have been addressed

Reviewer #2: All comments have been addressed

**********

2. Is the manuscript technically sound, and do the data support the conclusions?

Reviewer #1: Partly

Reviewer #2: Yes

**********

3. Has the statistical analysis been performed appropriately and rigorously?

Reviewer #1: Yes

Reviewer #2: Yes

**********

4. Have the authors made all data underlying the findings in their manuscript fully available?

The PLOS Data policy

Reviewer #1: Yes

Reviewer #2: Yes

**********

5. Is the manuscript presented in an intelligible fashion and written in standard English?

Reviewer #1: Yes

Reviewer #2: Yes

**********

Reviewer #1: Reviewe 1 had requested a demographics table to be added, Although the authours addressed the comment with a statement and not a in a table format. I accept that they have satisfied the reviewer's comment.

Reviewer #2: I have reviewed the revised manuscript, and the authors have adequately addressed most of my previous comments. The manuscript has been substantially improved. However, an additional clarification is needed regarding Comment 2.3.

The rationale for selecting the kidney transplant cohort has been clarified. However, the Discussion still does not explicitly acknowledge the absence of transplant-related clinical outcome analyses. Please add this as a limitation and clarify that the present findings do not establish transplant-specific clinical associations. In this context, the statement regarding “risk stratification” in the Conclusion should also be tempered accordingly.

**********

what does this mean?). If published, this will include your full peer review and any attached files.

If you choose “no”, your identity will remain anonymous but your review may still be made public.

**Do you want your identity to be public for this peer review?**  For information about this choice, including consent withdrawal, please see our Privacy Policy

Reviewer #1: No

Reviewer #2: No

**********

[NOTE: If reviewer comments were submitted as an attachment file, they will be attached to this email and accessible via the submission site. Please log into your account, locate the manuscript record, and check for the action link "View Attachments". If this link does not appear, there are no attachment files.]

To ensure your figures meet our technical requirements, please review our figure guidelines: https://journals.plos.org/plosone/s/figures

You may also use PLOS’s free figure tool, NAAS, to help you prepare publication quality figures: https://journals.plos.org/plosone/s/figures#loc-tools-for-figure-preparation.

NAAS will assess whether your figures meet our technical requirements by comparing each figure against our figure specifications.

# Author comment: Author response to Decision Letter 2

**DOI:** 10.1371/journal.pone.0360632.r005

27 Aug 2026

Journal Requirements:

Comment J.1.

If the reviewer comments include a recommendation to cite specific previously published works, please review and evaluate these publications to determine whether they are relevant and should be cited. There is no requirement to cite these works unless the editor has indicated otherwise.

Please review your reference list to ensure that it is complete and correct. If you have cited papers that have been retracted, please include the rationale for doing so in the manuscript text, or remove these references and replace them with relevant current references. Any changes to the reference list should be mentioned in the rebuttal letter that accompanies your revised manuscript. If you need to cite a retracted article, indicate the article’s retracted status in the References list and also include a citation and full reference for the retraction notice.

Response J.1

We thank the Editorial Office for these important recommendations regarding the verification of the reference list. In response, we manually reviewed the references cited in the revised manuscript and checked their publication status and bibliographic information.

During this review, we identified two references associated with a published corrigendum/erratum. We carefully examined the corresponding correction notices and confirmed that the reported corrections concern minor details and do not affect the findings, interpretation, or specific information for which these articles were cited in our manuscript. Therefore, we consider these references to remain relevant and appropriate for the purposes for which they are cited.

For transparency, the DOI of the corresponding corrigendum/erratum notices will be provided below:

1. Reference [9] – Al Naqbi H, Mawart A, Alshamsi J, Al Safar H, Tay GK. Major histocompatibility complex (MHC) associations with diseases in ethnic groups of the Arabian Peninsula. Immunogenetics. 2021;73: 131–152. doi:10.1007/s00251-021-01204-x

2. Reference [28] – O’Donnell TJ, Rubinsteyn A, Laserson U. MHCflurry 2.0: Improved Pan-Allele Prediction of MHC Class I-Presented Peptides by Incorporating Antigen Processing. Cell Syst. 2020;11: 42-48.e7. doi:10.1016/j.cels.2020.06.010

Neither of these articles has been identified as retracted. Accordingly, no references were removed on the basis of retraction. We have retained the two references because the published corrections do not alter the scientific rationale or conclusions supporting their citation in the present manuscript.

Reviewer #1:

Comment 1.1.

Reviewe 1 had requested a demographics table to be added, Although the authours addressed the comment with a statement and not a in a table format. I accept that they have satisfied the reviewer's comment.

Response 1.1.

We thank the reviewer for confirming that the addition of the demographic information adequately addresses the previous comment. We appreciate the reviewer’s consideration of this revision and are pleased that the information provided in the revised manuscript was considered sufficient to address the concern.

Reviewer #2:

Comment 2.1.

I have reviewed the revised manuscript, and the authors have adequately addressed most of my previous comments. The manuscript has been substantially improved. However, an additional clarification is needed regarding Comment 2.3.

The rationale for selecting the kidney transplant cohort has been clarified. However, the Discussion still does not explicitly acknowledge the absence of transplant-related clinical outcome analyses. Please add this as a limitation and clarify that the present findings do not establish transplant-specific clinical associations. In this context, the statement regarding “risk stratification” in the Conclusion should also be tempered accordingly.

Response 2.1.

We thank the reviewer for this important clarification. We agree that, although the rationale for selecting the kidney transplant cohort has now been clarified, the manuscript should explicitly acknowledge that transplant-related clinical outcomes were not analyzed. We have therefore added a specific statement in the Discussion identifying the absence of transplant-related clinical and virological outcome analyses as a limitation. We now clarify that the transplant cohort was used primarily as a source of HLA immunogenetic data and that the present findings do not establish associations between HLA variants, predicted HCMV antigen presentation, and transplant-specific clinical outcomes.

In addition, we have tempered the wording regarding “risk stratification” in the Conclusion and clarified that the predicted antigen-presentation profiles may inform future HLA-informed immune-monitoring studies, but should not be interpreted as evidence of transplant-specific risk stratification or clinical associations. We have also aligned the Abstract Conclusion with this clarification to ensure consistency throughout the manuscript.

Attachment Submitted filename: Reviewer Letter (3).docx

# Editor report: Decision Letter 2

**DOI:** 10.1371/journal.pone.0360632.r006

**Authors:** Maemu Gededzha

29 Sep 2026

Dear Dr. Carrasco

Thank you for submitting your manuscript to PLOS One. After careful consideration, we feel that it has merit but does not fully meet PLOS One’s publication criteria as it currently stands. Therefore, we invite you to submit a revised version of the manuscript that addresses the points raised during the review process.

Please submit your revised manuscript by 30/09/2026. If you will need more time than this to complete your revisions, please reply to this message or contact the journal office at plosone@plos.org. When you're ready to submit your revision, log on to https://www.editorialmanager.com/pone/ and select the 'Submissions Needing Revision' folder to locate your manuscript file.

- A letter that responds to each point raised by the academic editor and reviewer(s). You should upload this letter as a separate file labeled 'Response to Reviewers'.
- A marked-up copy of your manuscript that highlights changes made to the original version. You should upload this as a separate file labeled 'Revised Manuscript with Track Changes'.
- An unmarked version of your revised paper without tracked changes. You should upload this as a separate file labeled 'Manuscript'.

If you would like to make changes to your financial disclosure, please include your updated statement in your cover letter. Guidelines for resubmitting your figure files are available below the reviewer comments at the end of this letter.

If applicable, we recommend that you deposit your laboratory protocols in protocols.io to enhance the reproducibility of your results. Protocols.io assigns your protocol its own identifier (DOI) so that it can be cited independently in the future. For instructions see: https://journals.plos.org/plosone/s/submission-guidelines#loc-laboratory-protocols. Additionally, PLOS One offers an option for publishing peer-reviewed Lab Protocol articles, which describe protocols hosted on protocols.io. Read more information on sharing protocols at https://plos.org/protocols?utm_medium=editorial-email&utm_source=authorletters&utm_campaign=protocols.

As the corresponding author, your ORCID iD is verified in the submission system and will appear in the published article. PLOS supports the use of ORCID, and we encourage all coauthors to register for an ORCID iD and use it as well. Please encourage your coauthors to verify their ORCID iD within the submission system before final acceptance, as unverified ORCID iDs will not appear in the published article. *Only*  the individual author can complete the verification step; PLOS staff *cannot*  verify ORCID iDs on behalf of authors.

We look forward to receiving your revised manuscript.

Kind regards,

Maemu Petronella Gededzha, Ph.D

Academic Editor

PLOS One

Journal Requirements:

If the reviewer comments include a recommendation to cite specific previously published works, please review and evaluate these publications to determine whether they are relevant and should be cited. There is no requirement to cite these works unless the editor has indicated otherwise.

Please review your reference list to ensure that it is complete and correct. If you have cited papers that have been retracted, please include the rationale for doing so in the manuscript text, or remove these references and replace them with relevant current references. Any changes to the reference list should be mentioned in the rebuttal letter that accompanies your revised manuscript. If you need to cite a retracted article, indicate the article’s retracted status in the References list and also include a citation and full reference for the retraction notice.

Additional Editor Comments (if provided):

Address the outstanding comments from reviewer 2

[Note: HTML markup is below. Please do not edit.]

Reviewers' comments:

[NOTE: If reviewer comments were submitted as an attachment file, they will be attached to this email and accessible via the submission site. Please log into your account, locate the manuscript record, and check for the action link "View Attachments". If this link does not appear, there are no attachment files.]

To ensure your figures meet our technical requirements, please review our figure guidelines: https://journals.plos.org/plosone/s/figures

You may also use PLOS’s free figure tool, NAAS, to help you prepare publication quality figures: https://journals.plos.org/plosone/s/figures#loc-tools-for-figure-preparation.

NAAS will assess whether your figures meet our technical requirements by comparing each figure against our figure specifications.

# Editor report: Acceptance letter

**DOI:** 10.1371/journal.pone.0360632.r007

**Authors:** Maemu Gededzha

PONE-D-26-09386R2

PLOS One

Dear Dr. Carrasco,

I'm pleased to inform you that your manuscript has been deemed suitable for publication in PLOS One. Congratulations! Your manuscript is now being handed over to our production team.

At this stage, our production department will prepare your paper for publication. This includes ensuring the following:

* All references, tables, and figures are properly cited

* All relevant supporting information is included in the manuscript submission,

* There are no issues that prevent the paper from being properly typeset

You will receive further instructions from the production team, including instructions on how to review your proof when it is ready. Please keep in mind that we are working through a large volume of accepted articles, so please give us a few days to review your paper and let you know the next and final steps.

Lastly, if your institution or institutions have a press office, please let them know about your upcoming paper now to help maximize its impact. If they'll be preparing press materials, please inform our press team within the next 48 hours. Your manuscript will remain under strict press embargo until 2 pm Eastern Time on the date of publication. For more information, please contact onepress@plos.org.

You will receive an invoice from PLOS for your publication fee after your manuscript has reached the completed accept phase. If you receive an email requesting payment before acceptance or for any other service, this may be a phishing scheme. Learn how to identify phishing emails and protect your accounts at https://explore.plos.org/phishing.

If we can help with anything else, please email us at customercare@plos.org.

Thank you for submitting your work to PLOS One and supporting open access.

Kind regards,

PLOS One Editorial Office Staff

on behalf of

Dr. Maemu Petronella Gededzha

Academic Editor

PLOS One
